# Influence of bone condition on implant placement accuracy with computer-guided surgery

**DOI:** 10.1186/s40729-020-00249-z

**Published:** 2020-09-20

**Authors:** Ramadhan Hardani Putra, Nobuhiro Yoda, Masahiro Iikubo, Yoshihiro Kataoka, Kensuke Yamauchi, Shigeto Koyama, Upul Cooray, Eha Renwi Astuti, Tetsu Takahashi, Keiichi Sasaki

**Affiliations:** 1 Division of Advanced Prosthetic Dentistry, Tohoku University Graduate School of Dentistry, 4-1, Seiryo-machi, Sendai, Miyagi 980-8575 Japan; 2 Department of Dentomaxillofacial Radiology, Faculty of Dental Medicine, Universitas Airlangga, Surabaya, Indonesia; 3 Division of Oral Diagnosis, Tohoku University Graduate School of Dentistry, Sendai, Japan; 4 Division of Oral and Maxillofacial Surgery, Tohoku University Graduate School of Dentistry, Sendai, Japan; 5 Maxillofacial Prosthetics Clinic, Tohoku University Hospital, Sendai, Japan; 6 Division of International and Community Oral Health, Tohoku University Graduate School of Dentistry, Sendai, Japan

**Keywords:** Mandible, Dental implant, Computer-assisted surgery, Computed tomography

## Abstract

**Background:**

The impact of the jaw bone condition, such as bone quantity and quality in the implant placement site, affecting the accuracy of implant placement with computer-guided surgery (CGS) remains unclear. Therefore, this study aimed to evaluate the influence of bone condition, i.e., bone density, bone width, and cortical bone thickness at the crestal bone on the accuracy of implant placement with CGS.

**Methods:**

A total of 47 tissue-level implants from 25 patients placed in the posterior mandibular area were studied. Implant placement position was planned on the simulation software, Simplant® Pro 16, by superimposing preoperative computed tomography images with stereolithography data of diagnostic wax-up on the dental cast. Implant placement surgery was performed using the surgical guide plate to reflect the planned implant position. The post-surgical dental cast was scanned to determine the position of the placed implant. Linear and vertical deviations between planned and placed implants were calculated. Deviations at both platform and apical of the implant were measured in the bucco-lingual and mesio-distal directions. Intra- and inter-observer variabilities were calculated to ensure measurement reliability. Multiple linear regression analysis was employed to investigate the effect of the bone condition, such as density, width, and cortical bone thickness at the implant site area, on the accuracy of implant placement (*α* = 0.05).

**Result:**

Intra- and inter-observer variabilities of these measurements showed excellent agreement (intra class correlation coefficient ± 0.90). Bone condition significantly influenced the accuracy of implant placement using CGS (*p < 0.05*). Both bone density and width were found to be significant predictors.

**Conclusions:**

Low bone density and/or narrow bucco-lingual width near the alveolar bone crest in the implant placement site might be a risk factor influencing the accuracy of implant placement with CGS.

## Background

Implant placement with computer-guided surgery (CGS) has been widely used in the current implant dentistry practice. Specific computer software for implant placement planning can support clinicians to decide an ideal implant position based on imported three-dimensional computer tomography (CT) data of bone condition together with a scanned diagnostic wax-up model of the superstructure. Thereafter, the implant surgical guide plate is manufactured based on the virtual planning of the software, which can assist the surgical implant procedure resulting in a shorter duration of surgery and minimal discomfort to the patient [1–3].

Several studies regarding the accuracy of implant placement with CGS have reported that there can be some deviation between planned and placed implant positions [4–6]. A recent systematic review has demonstrated that the total mean deviation of 1.2 mm (1.04–1.44 mm) at the implant platform level and 1.4 mm (1.28–1.58 mm) at the implant apex level was found in the case of implant placement with CGS [4]. The deviation is influenced by several clinical and computational errors that could occur during the examination, planning, and surgical procedure [7]. Thus, to minimize the deviation in the implant placement with CGS, the factors related to these errors should be recognized.

So far, several reports revealed that some clinical factors such as the surgical technique, guide type, and implant position can affect the accuracy of the implant placement with CGS [8–10]. Jaw bone condition might be one of the essential factors that need to be assessed especially in the preoperative implant placement planning procedures [11] and possibly could affect the accuracy of the implant placement with CGS [8, 12]. Computerized tomography images can provide three-dimensional geometric and quantitative data of the jaw bone condition and also the bone mineral density which was determined based on the Hounsfield unit (HU) value [11, 13]. However, the impact of the jaw bone condition, such as bone quantity and quality in the implant placement site, affecting the accuracy of implant placement with CGS remains unclear.

This study was done to analyze the influence of bone condition on the accuracy of implant placement with CGS. Bone density, bone width, and cortical bone thickness at the crestal bone were investigated as influencing factors on implant placement accuracy.

## Material and methods

### Study design and sample selection

This study protocol was approved by the research ethics committee of Tohoku University Graduate School of Dentistry (reference number: 23-7). The subjects of this retrospective study were the patients who underwent dental implant treatment at the Dental Implant Centre, Tohoku University Hospital, Japan, between 2013 and 2018. For evaluating the accuracy of implant placement, the clear reference of the placed implant was essential. The tissue-level implants which have clear implant collar visibility as a reference were thus suitable for this analysis. As the tissue-level implants were used mainly in the mandibular posterior sites, which have different bone condition compared to the maxillary site, this study only focused on the mandibular posterior sites. Written informed consent was obtained from each patient. Patients who underwent bone augmentation and had some bone disease related to the jaw were excluded from this study. Thus, according to the selection criteria, a total number of 47 implants from 25 patients were included in this study.

### Clinical procedure

For radiographic examination, multi-detector CT scanning (Somatom Emotion 6, Siemens, Erlangen, Germany) was performed before implant placement for bone evaluation and implant planning for each patient. The impression of both dental arches of each patient was made using an alginate impression material (Aroma Fine Plus, GC, Tokyo, Japan), and a diagnostic wax-up simulating the prosthetic treatment with ideal shape and occlusion on the missing tooth area was performed on the cast model. The setup cast models with diagnostic wax-up were scanned and converted to the surface tessellation language (STL) data in the Simplant® Guide System (Dentsply Sirona, Charlotte, NC, USA). Both CT data and the STL data were imported into the computer simulation software Simplant® Pro 16 (Dentsply Sirona, Charlotte, NC, USA) for virtually planning the optimal number, position, and size of the implant based on the ideal prostheses and the available bone. This decision was made through a discussion by a plurality of members from different departments, including a prosthodontist, dental surgeon, and periodontist, in the clinical meeting. After determination, the stereolithographic surgical guide plate was fabricated in the Simplant® Guide System.

All surgical procedures were performed by oral surgery specialists (Y.K., K.Y., T.T.) with more than 10 years of experience. Implant placement with CGS was performed under local anesthesia. Minimally invasive flap elevation was performed prior to the drilling procedure. Tooth-supported surgical guide plate was used during the initial drilling procedures, whereas the implants were inserted directly without the guide plate. This was followed by a tension-free closure around the healing abutment using non-resorbable nylon sutures (Soſtretch, GC, Tokyo, Japan). The impression of fabricating the implant superstructure was also performed around 2 months after the implant placement surgery, and the final implant prosthesis was set on the implant.

### Bone condition analysis

Bone density, bone width, and cortical bone thickness at the implant placement area were measured using the Simplant® Pro 16. The measurement was performed on the cross-sectional CT images taken before implant placement surgery.

**Fig. 1:**
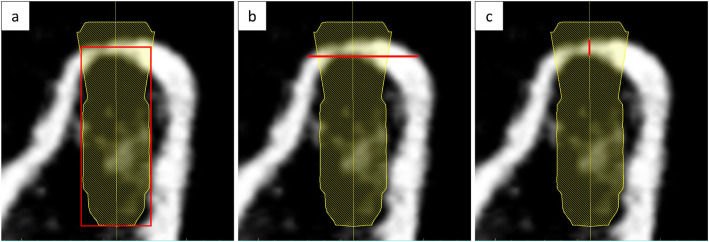
Bone condition analysis. **a** Bone density measurement. Red box indicates the measured area with an implant outer area as a reference. **b** Bone width measurement. Red bar indicates the measured area at 1 mm under the crest of alveolar bone. **c** Cortical bone thickness measurement. Red bar indicates the measured area parallel to the dental implant axis

Bone density (BD) was measured by drawing a rectangular region of interest including the cortical and cancellous bone area, which was referred to as the outer part of the virtually planned implant (Fig. 1a). Bone width (BW) was measured at 1 mm under the crest of the alveolar bone (Fig. 1b). Cortical bone thickness (CBT) was measured parallel to the centerline of the virtually placed implant (Fig. 1c). Further, these bone condition parameters were divided into two groups (Table 1) for the multiple regression analysis. BD was divided into two groups based on the mean of overall bone density, which was approximately 500 HU. Available BD classification (e.g., Misch [11] or Norton and Gamble classification [14]) could not be applied in our study because there were only a few numbers of samples classified in some groups. BW was divided according to the recommendation of minimum available BW by Misch [11], and CBT was divided based on the crestal cortical bone measured by Sugiura et al. [15].

**Table 1:** Categories of bone condition measured in the implant site area

Bone condition	Criteria
**Bone density**	
BD1	< 500 HU
BD2	> 500 HU
**Bone width**	
BW1	< 6 mm
BW2	> 6 mm
**Cortical bone thickness**	
CBT1	< 1.5 mm
CBT2	> 1.5 mm

*HU* Hounsfield units

### Implant placement accuracy measurement

**Fig. 2:**
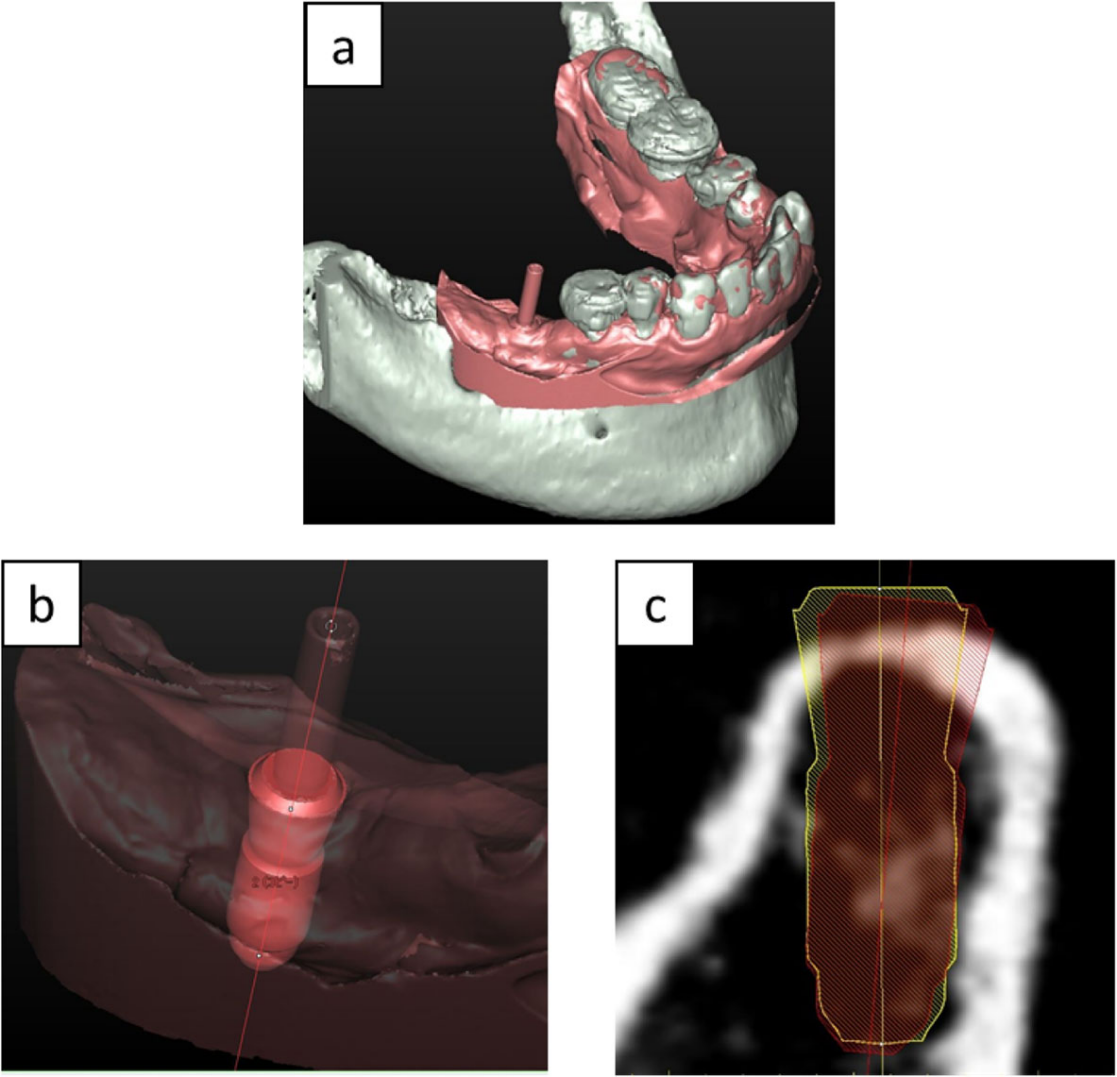
Assessment of placed implant position. **a** Matching digital data between preoperative planned computed tomography image (gray-colored model) and scanned postoperative impression (pink-colored model). **b** Assessing placed implant position using implant collar and guide pin as references. **c** Comparing planned (yellow-colored) and placed (red-colored) implant position

Implant placement accuracy was evaluated by comparing the position of the virtual planned implant and placed implant using the Simplant® Pro 16. Placed implant position was determined using the STL data obtained from the scanned post-surgical cast model, which was used for fabricating the final superstructure. When scanning the post-surgical cast model, the guide pin (Strauman AG, Basel, Switzerland) was included. Surface tessellation language data of the post-surgical cast model was imported into the Simplant® Pro 16 and was superimposed with the CT data, which contained the information of the planned implant position. The anterior tooth and the most posterior tooth on both sides were used as reference points for the superimposition (Fig. 2a). The reference point was set on the clear incisal area of the anterior natural tooth, while the tooth cusp was set as a reference point of a natural posterior tooth. This registration procedure was then confirmed by inspecting the outline of aligned digital data. Next, a virtual implant, which was duplicated from the planned implant, was placed into the post-surgical STL model and was superimposed with the placed implant using the implant neck collar area as a reference. The implant guide pin, which was scanned together with the post-surgical cast model, was also used as a reference for correcting the implant angulation (Fig. 2b). Finally, the positional accuracy was evaluated by comparing the virtually planned position with that of the placed implant (Fig. 2c).

**Fig. 3:**
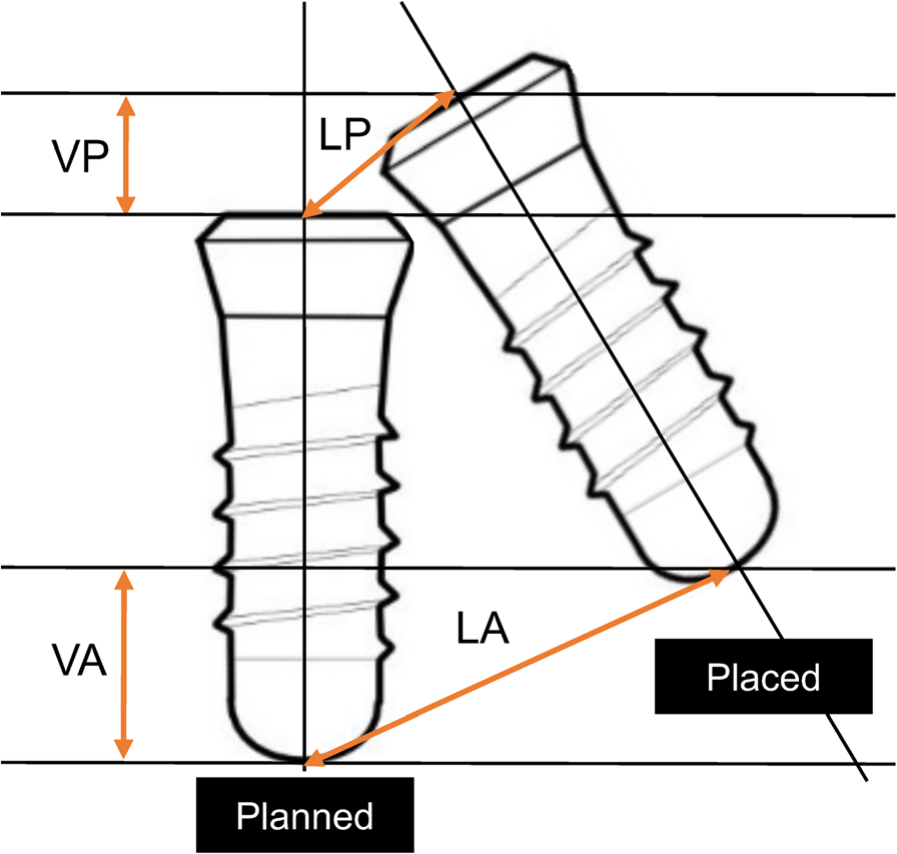
Parameters for implant deviation measurement. VP, vertical deviation at implant platform (mm); VA, vertical deviation at implant apex (mm); LP, linear deviation at implant platform; LA, linear deviation at implant apex (mm)

**Fig. 4:**
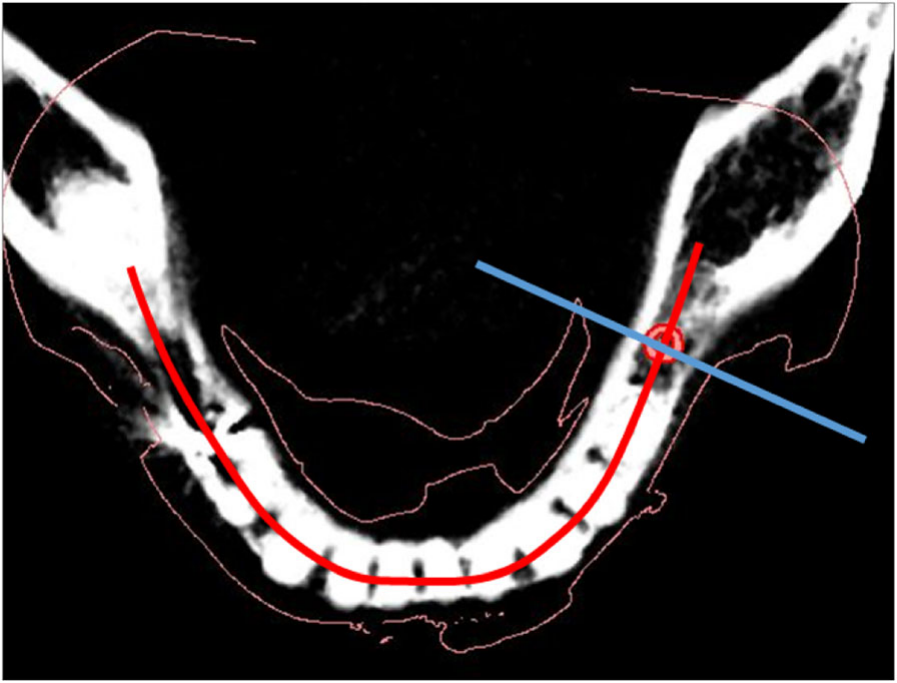
Bucco-lingual section plane (red line) and mesio-distal section plane (blue line)

The implant placement accuracy was measured according to the following parameters (Fig. 3): (1) linear deviation at the implant platform (LP), (2) linear deviation at the implant apical (LA), (3) vertical deviation at the implant platform (VP), and (4) vertical deviation at the implant apical (VA). The reference point of the implant platform and apical was set to the intersection point between the implant axis and the most coronal/apical part of the implant. Both LP and LA were measured using the bucco-lingual (BL) plane and mesio-distal (MD) plane. BL plane was a plane of mandibular arch line, and MD plane was the plane perpendicular to the mandibular arch line in the implant site area (Fig. 4). The cross-sectional BL and MD two-dimensional images were employed instead of three-dimensional distance for clear clinical understanding. The implant position orthogonal projection validation method [16] was referred for this analysis. VP and VA were measured only in the BL direction as it had an identical deviation in the MD direction.

To ensure the reliability of these methods, intra- and inter-observer variability were calculated. For intra-observer reliability, each implant placement accuracy was measured twice at an interval of 4 weeks. Inter-observer reliability was evaluated by three observers, two radiologists (R.H.P. and M.I.) and an implantologist (N.Y.). To estimate intra- and inter-observer precision, technical error of measurement (TEM) of inter- and intra-observer was calculated using the formula (∑*D*^2^/2*N*)^0.5^, where *D* is the difference between the two measurements and *N* is the number of samples [17, 18].

### Statistical analysis

The statistical analysis was carried out using SPSS (IBM SPSS Statistics for Windows version 25.0, IBM Corp., NY, USA). Intra- and inter-observer measurement reliability were assessed with single-measured, intraclass correlation coefficient (ICC) with absolute agreement and two-way mixed-effect model.

Correlation between each bone condition and implant accuracy parameters was tested with the Pearson correlation coefficient. Further, multiple linear regression analysis was employed to analyze the correlation between combined bone conditions on each implant accuracy parameters. Bone density, bone width, and cortical bone thickness represented in Table 1 were used as independent variables for multiple linear regression analysis. BL-LP, BL-LA, MD-LP, MD-LA, VP, and VA were used as dependent variables. *p* values less than 0.05 were considered significant.

## Results

This retrospective study evaluated the implant position of 8 male and 17 female patients. The mean age was 64 ± 8.1 years (range, 51–81 years). The distribution of implant dimension and position is represented in Table 2. Various implant dimensions placed in the premolar and/or molar area were observed in this study.

**Table 2:** Distribution of implant according to the dimension and region of insertion

	Total implants
**Implant dimension (diameter × length)**	
3.3 mm × 10 mm	2
4.1 mm × 6 mm	2
4.1 mm × 8 mm	7
4.1 mm × 10 mm	25
4.1 mm × 12 mm	5
4.8 mm × 8 mm	1
4.8 mm × 10 mm	4
4.8 mm × 12 mm	1
**Implant position**	
Premolar region	14
Molar region	33

The mean, median, standard deviation, and minimum and maximum value of the implant placement accuracy parameters in each bone condition group are shown in Table 3. LP and LA were further divided into deviation in the BL and MD directions. Because both vertical deviations had a similar result between BL and MD direction, only the result in the BL direction was presented.

**Table 3:** Descriptive statistics of implant placement accuracy parameters on each bone condition groups

	Bone density		Bone width		Cortical bone thickness	
	BD1	BD2	BW1	BW2	CT1	CT2
*N*	24	23	36	11	33	14
**BL-LP (mm)**						
Mean	1.63	1.30	1.56	1.16	1.53	1.32
Median	1.43	1.12	1.43	0.92	1.35	1.11
SD	0.84	0.71	0.75	0.86	0.84	0.66
Min	0.56	0.27	0.42	0.27	0.27	0.55
Max	3.32	2.66	3.32	2.98	3.32	2.31
**BL-LA (mm)**						
Mean	1.94	1.56	1.88	1.34	1.46	1.32
Median	1.69	1.63	1.86	1.17	1.30	1.43
SD	0.95	0.83	0.90	0.82	0.87	0.63
Min	0.53	0.25	0.25	0.42	0.24	0.3
Max	4.23	3.01	4.23	2.9	3.24	2.32
**MD-LP (mm)**						
Mean	1.63	1.19	1.52	1.08	1.84	1.54
Median	1.44	1.30	1.45	0.71	1.63	1.67
SD	0.84	0.71	0.75	0.91	0.94	0.82
Min	0.38	0.24	0.24	0.38	0.26	0.25
Max	3.24	2.65	3.24	2.93	4.23	2.82
**MD-LA (mm)**						
Mean	2.40	1.39	1.93	1.82	1.98	1.72
Median	2.19	1.32	1.82	1.14	1.70	1.83
SD	0.94	0.74	0.91	1.22	1.03	0.86
Min	0.95	0.45	0.45	0.5	0.45	0.48
Max	4.04	3.41	4.04	3.67	4.04	3.34
**VP (mm)**						
Mean	1.50	0.96	1.34	0.88	1.31	1.06
Median	1.29	0.81	1.30	0.53	1.20	0.80
SD	0.85	0.78	0.81	0.94	0.92	0.67
Min	0.42	0.08	0.08	0.08	0.08	0.25
Max	3.25	2.65	3.25	2.79	3.25	2.25
**VA (mm)**						
Mean	1.68	0.96	1.43	0.98	1.43	1.09
Median	1.39	0.86	1.39	0.50	1.36	0.93
SD	0.90	0.84	0.88	1.05	0.96	0.86
Min	0.41	0.01	0.01	0.02	0.01	0.01
Max	3.46	2.68	3.46	3.26	3.46	2.65

*BL* bucco-lingual, *LP* linear deviation at the implant platform, *LA* linear deviation at the implant apical, *MD* Mesio-distal, *VP* vertical deviation at the implant platform, *VA* vertical deviation at the implant apical

Table 4 shows the intra- and inter-observer measurement reliabilities and TEM related to all implant placement accuracy parameters. Intra-observer measurement on all implant accuracy parameters showed excellent agreement (ICC > 0.90) with TEMs about 0.10 mm. Inter-observer measurement also showed good and excellent agreement (0.87 > ICC > 0.95) between three observers with TEMs about 0.20 mm.

**Table 4:** Intra- and inter-observer measurement reliabilities and TEM for implant placement accuracy parameters

Observer	BL-LP	MD-LP	BL-LA	MD-LA	VP	VA
**Intra-observer**	0.986 (0.095)	0.990 (0.080)	0.983 (0.114)	0.993 (0.080)	0.991 (0.079)	0.991 (0.089)
**Inter-observer**						
R.H.P	NA	NA	NA	NA	NA	NA
N.Y.	0.937 (0.194)	0.909 (0.223)	0.936 (0.224)	0.904 (0.243)	0.896 (0.256)	0.911 (0.268)
M.I.	0.885 (0.238)	0.831 (0.290)	0.968 (0.163)	0.889 (0.261)	0.898 (0.241)	0.920 (0.241)
**Average**	**0.911 (0.216)**	**0.870 (0.257)**	**0.952 (0.193)**	**0.897 (0.252)**	**0.897 (0.249)**	**0.916 (0.255)**

Measurements are made by R.H.P (Ramadhan H. Putra), N.Y. (Nobuhiro Yoda), and M.I. (Masahiro Iikubo). Table entries are intraclass correlation coefficient of intra- and inter-observer measurement using single-measure, absolute agreement, and two-way mixed model; TEM in millimeters (in parentheses)

*NA* not applicable, *BL* bucco-lingual, *LP* linear deviation at the implant platform, *LA* linear deviation at the implant apical, *MD* mesio-distal, *VP* vertical deviation at the implant platform, *VA* vertical deviation at the implant apical

**Fig. 5:**
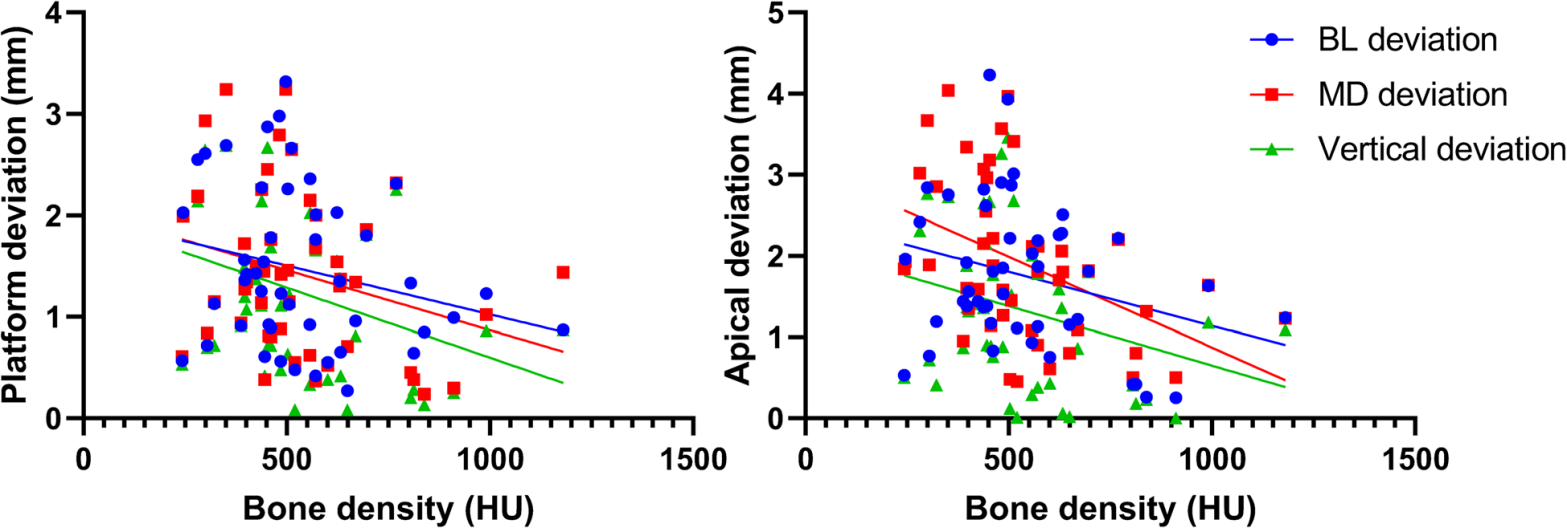
Scatter plot showing a linear correlation between bone density and implant deviation. Left: deviation at the implant platform; right: deviation at the implant apical

**Fig. 6:**
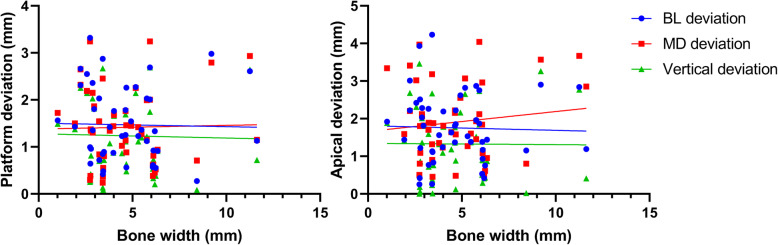
Scatter plot showing a linear correlation between bone width and implant deviation. Left: deviation at the implant platform; right: deviation at the implant apical

**Fig. 7:**
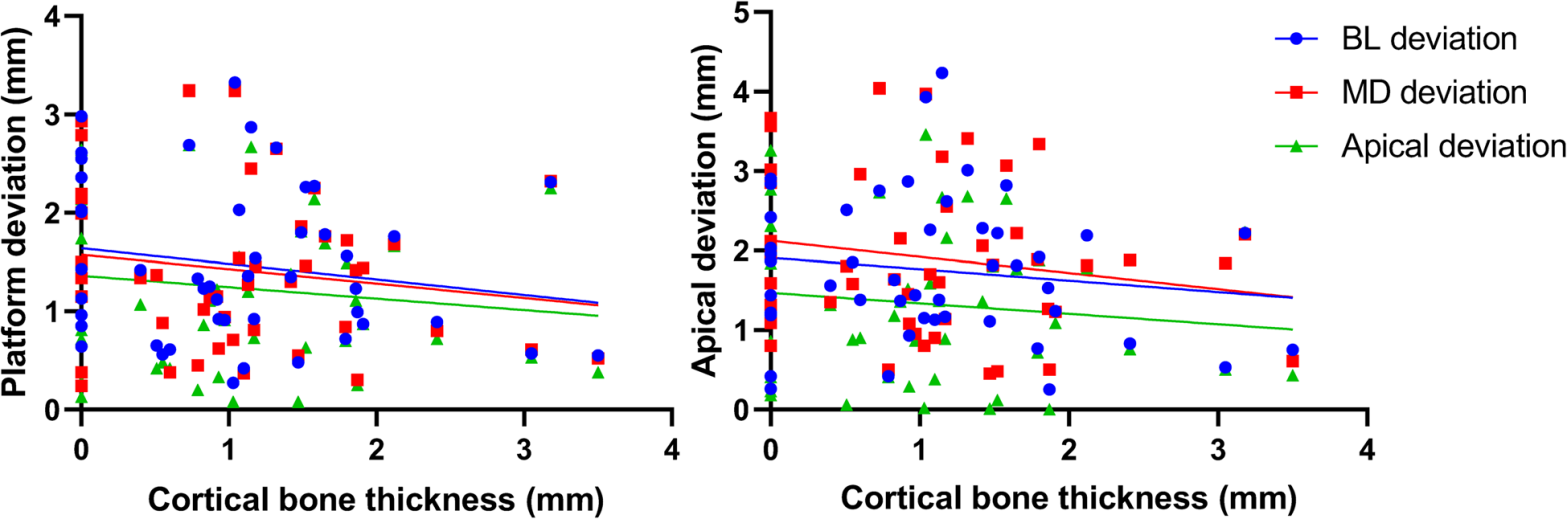
Scatter plot showing a linear correlation between cortical bone thickness and implant deviation. Left: deviation at the implant platform; right: deviation at the implant apical

The distribution of placement accuracy parameters related to each bone condition (BD, BW, and CBT) is shown in Figs. 5, 6, and 7. There were significant negative correlations between bone density and MD-LA (*r* = − 0.44; *p* = 0.002), VP (*r* = − 0.32; *p* = 0.03), and VA (*r* = − 0.31; *p* = 0.04) (Fig. 5). No statistically significant correlation was found between bone width and cortical bone thickness on any deviation parameters (Figs. 6 and 7). In Fig. 6, 1- and 2-mm bone width were observed when the bone width was measured at 1 mm under the crest of the alveolar bone. Those conditions were especially observed in the tapered alveolar crest case. In Fig. 7, the cortical bone thickness in some samples were not present. This condition appeared in some cases because the post-extraction socket was not completely healed when pre-surgical CT examinations were performed.

The result of multiple regression analysis of implant placement accuracy using bone condition as a dependent variable was shown in Table 5. Bone condition significantly influenced implant placement accuracy in terms of BL-LA, MD-LA, VP, and VA (*p* < 0.05). The highest *R*^2^ value was found in MD-LA (*R*^2^ = 0.31) which indicates 31% of the variation of implant deviation was explained by BD, BW, and CBT. The least deviation was found in MD-LA, which showed a 1.05 mm lesser deviation in the BD2 group than in the BD1 group after controlling the effect of BW and CBT. From all predictors, BD and BW have a significant effect on almost all implant placement accuracy parameters (*p* < 0.05).

**Table 5:** Multiple linear regression analysis of the correlation between bone condition and implant placement accuracy parameters

		BL-LP	MD-LP	BL-LA	MD-LA	VP	VA
Bone density	(B)	− 0.39	− 0.50*	− 0.46	− 1.05**	− 0.61*	− 0.79**
	(I)	[− 0.84, 0.06]	[− 0.95, − 0.05]	[− 0.97, 0.04]	[− 1.55, − 0.55]	[− 1.08, − 0.15]	[− 1.28, − 0.30]
Bone width	(B)	− 0.50	− 0.55	− 0.68*	− 0.33	− 0.61*	− 0.65*
	(I)	[− 1.04, 0.03]	[− 1.08, − 0.01]	[− 1.28, − 0.07]	[− 0.93, 0.27]	[− 1.16, − 0.05]	[− 1.23, − 0.06]
Cortical bone thickness	(B)	− 0.28	− 0.20	− 0.38	− 0.28	− 0.32	− 0.41
	(I)	[− 0.77, 0.21]	[− 0.69, 0.29]	[− 0.94, 0.17]	[− 0.83, 0.27]	[− 0.83, 0.19]	[− 0.95, 0.13]
*R*^2^		0.13	0.16	0.17	0.31	0.21	0.26
Overall *p* value		0.099	0.051	0.045	0.001	0.016	0.004

B: coefficient; 95% confidence intervals in brackets; confidence level *p < 0.05, **p < 0.01

*BL* bucco-lingual, *LP* linear deviation at the implant platform, *LA* linear deviation at the implant apical, *MD* mesio-distal, *VP* vertical deviation at the implant platform, *VA* vertical deviation at the implant apical

## Discussion

Bone condition assessment is generally performed before implant placement surgery for assessing the implant number, size, and angulation. Three-dimensional CT images can provide accurate anatomical and geometrical information regarding the bone condition [11, 19]. In all subjects who participated in this study, multi-detector CT was taken for preoperative radiographic examination. Multi-detector CT can provide not only accurate information about the bone shapes but also the bone density data based on the HU value [20–22]. Our study analyzed bone width and cortical bone thickness as predictors. Bone width near the alveolar bone crest is generally assessed to select proper implant size and insertion site based on the available bone so that the bone surrounding the inserted implant could be well preserved, resulting in good primary implant stability [23]. While cortical bone thickness also correlates with the implant primary stability [24, 25], we included the parameters such as bone condition since it plays a role in successful dental implant treatment.

According to the results of this study, when each of the bone condition predictors was investigated as a single predictor of implant placement accuracy, only the bone density had a significant negative correlation on implant placement accuracy with CGS (Fig. 5). It means that higher implant deviation was observed in the low bone density condition. These findings concur with the previous study which reported that bone density had a highly negative correlation with an angular deviation of half-guided implant surgery [26]. Another study also reported the significant negative correlation between bone density and vertical deviation both in the implant platform and the apical area in mucosa-supported CGS [27].

Interestingly, when multiple bone condition predictors were considered, bone density, bone width, and cortical bone thickness significantly influenced the accuracy in implant placement with CGS. All bone conditions had a negative correlation, especially bone density and bone width which significantly influenced the implant placement accuracy. It means that the poor bone condition such as low bone density, narrow bone width, and/or thin cortical bone thickness has a higher risk of causing some deviation in implant placement. Based on our results, the poor bone condition had highly influenced the deviation at the implant apical. These findings might certify that keeping a safety zone of at least 2 mm is still critical to avoid surrounding anatomical structure damage near the implant apical even in the case of CGS [4, 5, 28].

As the bone density alone significantly influenced implant placement accuracy (Fig. 5), the deviation can be even higher if narrow bone width and thin cortical bone thickness are also co-existing. When all the parameters of the bone condition were considered, the implant placement accuracy was significantly influenced by the bone density around the planned implant placement site and the bone width at 1 mm under the crestal bone. As shown in the multiple regression results (Table 5), the highest deviation was found in the cases that the bone density was less than 500 HU bone after controlling the effect of bone width and cortical bone thickness. It should be noted that the surgical procedure followed was not a fully guided implant surgery, which means that the pilot guide template (Simplant®) was only used for the pilot drill of osteotomy. In the case of low bone density, the drilling trajectory might deviate toward the softer part of the bone after the second drilling procedure. In addition, if bone width is narrow at the crestal bone, the initial drilling point might also have deviated from the initially planned area resulting in higher deviation during implant placement. For this reason, our study concluded that higher implant placement deviation can occur in cases with poor bone condition, especially when both low bone density and narrow bone width are present. Clinically, a fully guided surgery protocol might be recommended to minimize the deviation. Two RCT studies comparing different guided surgery protocol also reported that fully guided surgery protocol showed better results in comparison with the cases using a surgical guide plate only at pilot drill [29, 30].

In this study, scanned post-surgical cast model data were used to determine the actual placed implant position. Most of the studies used postoperative radiographic imaging for evaluating the actual placed implant position [5–7, 12, 24, 26, 27, 29–31]. However, it might increase the patient’s additional irradiation exposure, which is not in accordance with the principle of as low as reasonably achievable. Moreover, determining the placed implant position was challenging in the case that the outline of the implant on the radiograph images was unclear and blurred [32, 33]. Avoiding the additional irradiation exposure, STL data of the post-surgical cast model were used in this study to confirm the actual placed implant position despite the lower accuracy owing to its manual procedures. Only tissue-level implants (Strauman AG, Basel, Switzerland) with clear visibility of implant collar were thus included in this study. To compensate for the reliability of our manual initiative method, intra- and inter-observer measurement reliability was tested. Consequently, excellent agreement (ICC ± 0.900) with 0.10–0.20 mm of TEM was demonstrated on intra- and inter-observer measurements. An intraoral scan data [32, 34] and optical scan data of dental cast [35] should be useful for confirming the postoperative implant position.

The implant placement accuracy with CGS is generally affected by cumulative factors from the clinical and technical errors, which arise during the examination, planning, laboratory process, and/or surgical procedures [8, 36, 37]. Various patterns of edentulous area in the posterior mandible, number of placed implants, and implant dimension, which can influence the accuracy outcome [10, 31, 38], were found in our study. However, the influences of those factors were not analyzed due to the limited number of subjects as well as the uneven distribution of those factors. The *R*^2^ value indicated that even in our highest significant model, only 31% of the implant deviation was explained by the bone condition. Low *R*^2^ value could mainly be a result of unmeasured possible confounding factors related to the implant deviation, such as errors during CT examination, impression taking, segmentation in planning software, production of surgical guide, and complicated implant surgery (e.g., patient limited mouth opening, swollen mucosa after local anesthesia, and tolerance of surgical instrument) [4, 36]. Those factors might coincide with poor bone condition and enlarged the range of implant deviation (Figs. 5, 6, and 7). Further studies with a larger sample size will be able to clarify the influence of not only bone condition but also other potential factors on implant placement accuracy. Even though CGS offers many advantages over conventional implant surgery, a better understanding of the extent to which bone condition can influence the implant placement accuracy is essential for clinicians despite the use of the computer-guided surgical template.

## Conclusion

Within the limitations of this study, the bone condition can influence the accuracy of implant placement with CGS. Low bone density and/or narrow bucco-lingual width near the alveolar bone crest in the implant placement site might be a risk factor that can lead to implant placement deviation. Therefore, the clinician should consider such bone conditions when performing implant placement surgery despite using CGS, especially in the case using a pilot drill surgical guide. Further well-designed studies with a larger sample size are required to confirm these findings.

## Data Availability

The datasets used and/or analyzed during the current study are available from the corresponding author on reasonable request.

## Abbreviations

CGS: Computer-guided surgery
CT: Computed tomography
BD: Bone density
BW: Bone width
CBT: Cortical bone thickness
STL: Surface tessellation language
BL: Bucco-lingual
MD: Mesio-distal
LP: Linear platform
LA: Linear apical
VP: Vertical platform
VA: Vertical apical
